# Diagnostic accuracy of SPECT/CT arthrography in patients with suspected aseptic joint prostheses loosening

**DOI:** 10.1186/s41824-021-00098-y

**Published:** 2021-02-28

**Authors:** Bo Bao, Crystal S. Liu, Edward C. O. Masson, Jonathan T. Abele

**Affiliations:** 1 Department of Radiology and Diagnostic Imaging, University of Alberta, 2A2.41 Walter C Mackenzie Health Sciences Centre, Edmonton, Alberta T6G 2B7 Canada; 2 Cumming School of Medicine, University of Calgary, Calgary, Alberta Canada; 3 Division of Orthopedic Surgery, University Alberta, Edmonton, Alberta Canada

**Keywords:** Total knee arthroplasty, Total hip arthroplasty, SPECT/CT, Loosening

## Abstract

**Purpose:**

To evaluate the diagnostic accuracy of SPECT/CT arthrography in patients with suspected aseptic prosthesis loosening following hip and knee arthroplasty.

**Methods:**

A retrospective review of 63 SPECT/CT arthrogram studies (36 knees and 27 hips) between February 1, 2013, and July 1, 2018, was conducted. All patients underwent clinical and radiologic evaluation as part of their assessment for persistent pain following hip and knee arthroplasty. The detection of tracer activity along the bone-prosthetic interface on SPECT/CT suggests aseptic loosening. Operative assessment as well as clinical/radiologic follow-up at a minimum of 1 year was used as the reference standard.

**Results:**

The sensitivity and specificity of SPECT/CT for detection of aseptic loosening was 6/7 (86%) and 55/56 (98%), respectively. This gives a positive predictive value (PPV) of 6/7 (86%), a negative predictive value (NPV) of 55/56 (98%), and a diagnostic accuracy of 61/63 (97%).

**Conclusion:**

SPECT/CT arthrography has a high diagnostic accuracy (97%) in the evaluation of loosening of both hip and knee arthroplasties in patients with persistent post-procedural pain.

## Introduction

Hip and knee arthroplasties are commonly performed procedures for the management of osteoarthritis (Katz et al. 2010; Ethgen et al. 2004). In the USA, over 7.2 million individuals have received hip and knee replacement surgeries (Maradit Kremers et al. 2015). The rate of joint replacement surgery is projected to increase further in the next few years (Singh et al. 2019). While hip and knee arthroplasties are successful in most patients, persistent pain is a common complication that affects up to 44% of patients with total hip arthroplasty and 27% of patients with total knee arthroplasty (Wylde et al. 2011; Piscitelli et al. 2013; Becker et al. 2011). Although post-surgical pain can be due to a variety of causes, aseptic loosening is the most common complication of hip and knee arthroplasty requiring major revision surgery (Sharkey et al. 2014). Hence, accurate detection of aseptic loosening is essential in guiding management decisions for persistent post-arthroplasty pain.

Aseptic loosening describes a failure in the integration of the bone and prosthesis that is not due to an infection (Sundfeldt et al. 2006). Instead, wear debris forms at the implant-bone interface and causes chronic inflammation. The end result is an osteolytic process that leads to loosening (Abu-Amer et al. 2007). Since progression of periprosthetic tissue destruction can be subtle, clinical diagnosis of aseptic loosening in patients is challenging. Initial workup typically involves a detailed history, clinical examination, and conventional radiographs during follow-up. For complex patients, adjunct imaging modalities such as bone scintigraphy, radionuclide arthrography, single photon-emission computed tomography/computed tomography (SPECT/CT), 18F-FDG-PET, and/or MRI can be used for detecting aseptic loosening (Lohmann et al. 2017). In this retrospective study, our goal was to confirm the accuracy of SPECT/CT arthrography in patients with clinically suspected hip or knee arthroplasty loosening.

## Materials and methods

### Data collection

This study is a retrospective analysis of all SPECT/CT arthrogram studies referred to our center between February 1, 2013, and July 1, 2018. During this period, a total of 94 SPECT/CT arthrogram studies were performed for the evaluation of persistent pain after hip and knee arthroplasty. Institutional ethics review board approval of our study protocol was obtained.

**Fig. 1:**
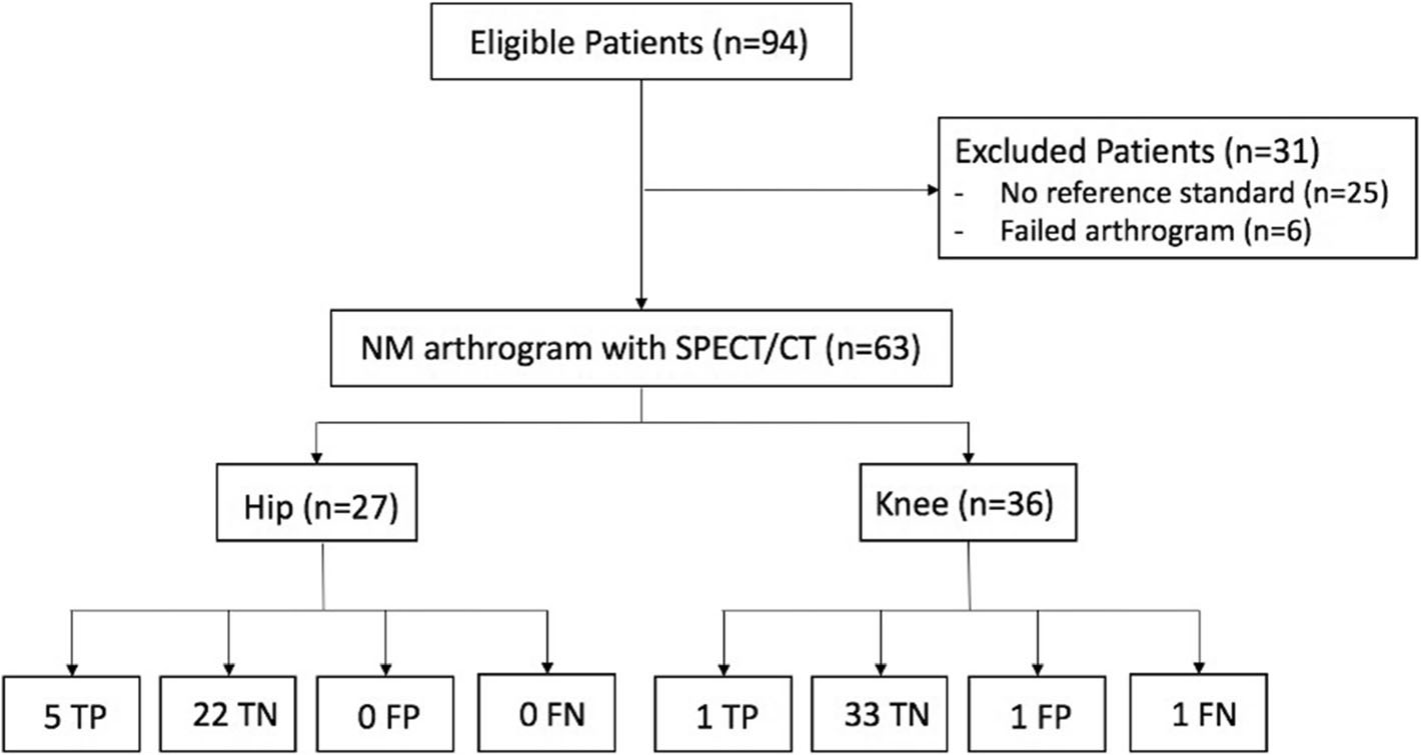
Study participants. TP, true positive; TN, true negative; FP, false positive; FN, false negative

Demographic information (age, gender), type of prosthesis (cemented vs non-cemented), radiographic diagnosis, clinical findings, and surgical reports were exported for analysis. All patients had an initial standard radiograph exam followed by SPECT/CT arthrogram as part of the diagnostic pathway. Patients were only included in the study if they were subsequently followed up by an orthopedic surgeon, had an operative revision, or a minimum of 1-year period of observation. As well, joint aspiration results during the follow-up period and after revision surgery were reviewed to exclude septic etiology. Six studies were excluded from analysis due to failed tracer injections as indicated by tracer activity seen outside the joint capsule on the SPECT/CT images (i.e., extracapsular injection despite fluoroscopic guidance). These patients were typically re-booked for a repeat study and the data from these repeats may be included in the data set. Twenty-five patients were excluded since no reference standard was available, either because they did not have subsequent operative assessment or no follow-up clinic data could be obtained. The remaining 63 patient studies were included for analysis (Fig. 1).

### Imaging procedure

A 22-gage needle was inserted into the joint space of interest under fluoroscopic guidance by an experienced interventional radiologist. This was approached via the inferolateral margin of femoral neck component for those with a prior hip arthroplasty or subpatellar joint space for those with a prior knee arthroplasty. Positioning within the joint space was confirmed with the injection of 2 mL of iodinated contrast (Omnipaque 300, GE Healthcare, Buckinghamshire, UK). Once confirmed, 1 mCi (37 MBq) of Tc-99m sulfur colloid in 2 mL of sterile saline was injected into the joint. The patient was then encouraged to ambulate for a minimum of 30 min before being imaged. If unable to ambulate, a minimum of 1-h delay was required before imaging.

Initially, planar images were obtained in anterior, posterior, and lateral positions (128 × 128 matrix, low-energy high-resolution collimator, 10 min per acquisition). SPECT/CT images were then acquired using a hybrid SPECT/CT system equipped with a low-energy, high-resolution collimator (Philips Precedence, Best, the Netherlands; Philips Brightview, Best, the Netherlands; or Siemens Symbia, Munich, Germany). The CT parameters were 80 mA, 120–130 kV, 512 × 512 matrix size, and 1–5-mm slice thickness. SPECT/CT was performed with a matrix size of 128 × 128, 1.0 zoom, 20 s per frame, and 120 frames at 3° intervals.

All images were reconstructed using vendor-recommended iterative reconstruction algorithms. Decay correction and attenuation correction were both applied. No post-reconstruction filter was applied.

**Fig. 2:**
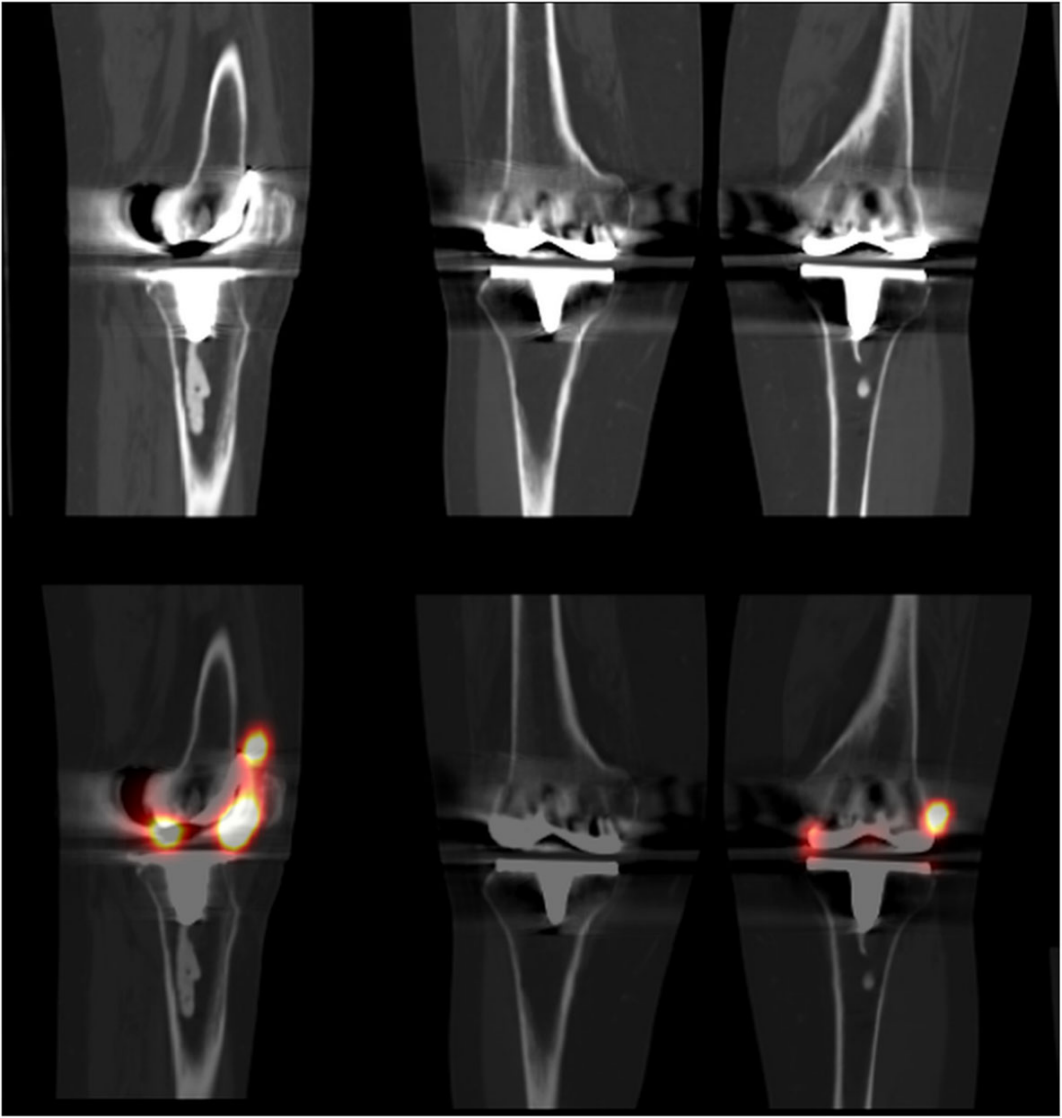
Normal left knee total arthroplasty. Sagittal (left) and coronal (right) imaging with CT (top row) and fused SPECT/CT (bottom row) images showing normal tracer activity within the joint space

**Fig. 3:**
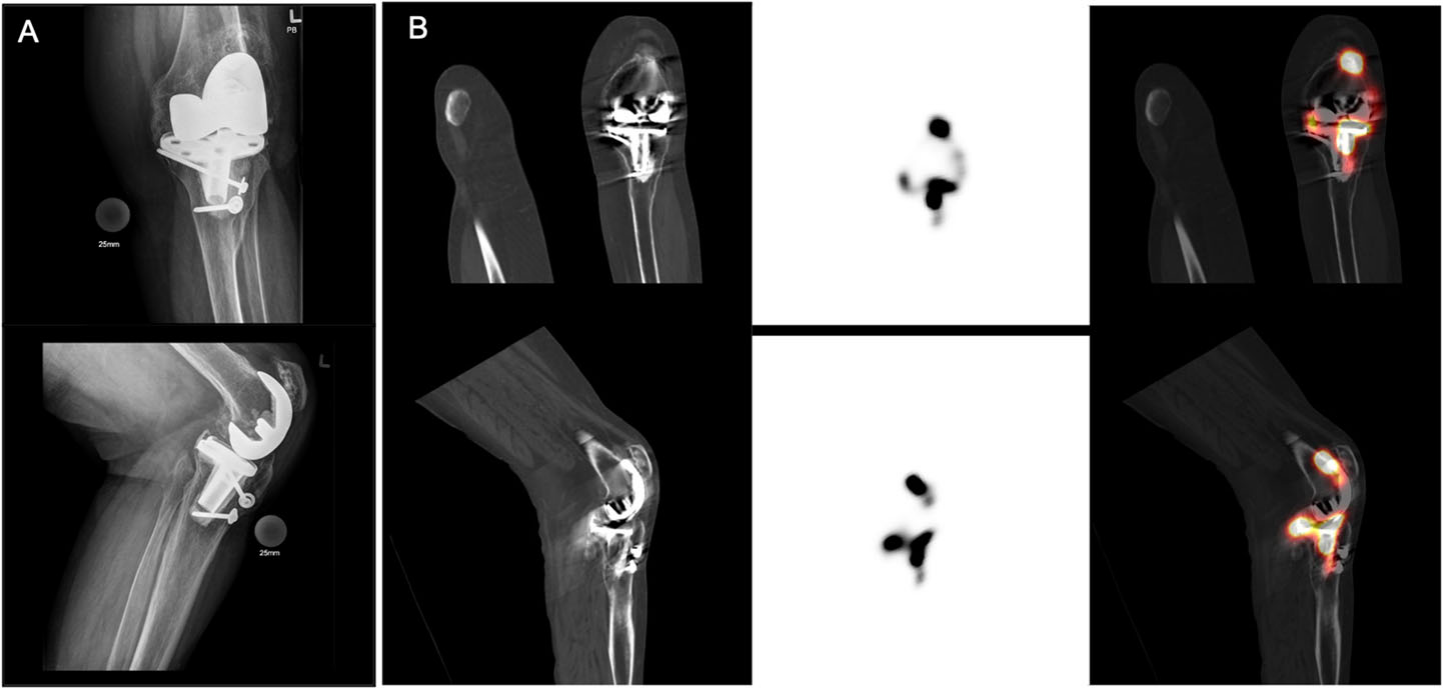
Tibial component loosening of a left knee total arthroplasty. The radiograph images demonstrate no evidence of loosening (first column). Coronal (top) and sagittal CT (second column), SPECT (third column), and fused SPECT/CT (fourth column) images showing abnormal tracer activity along the bone-prosthetic interface of the tibial component

**Fig. 4:**
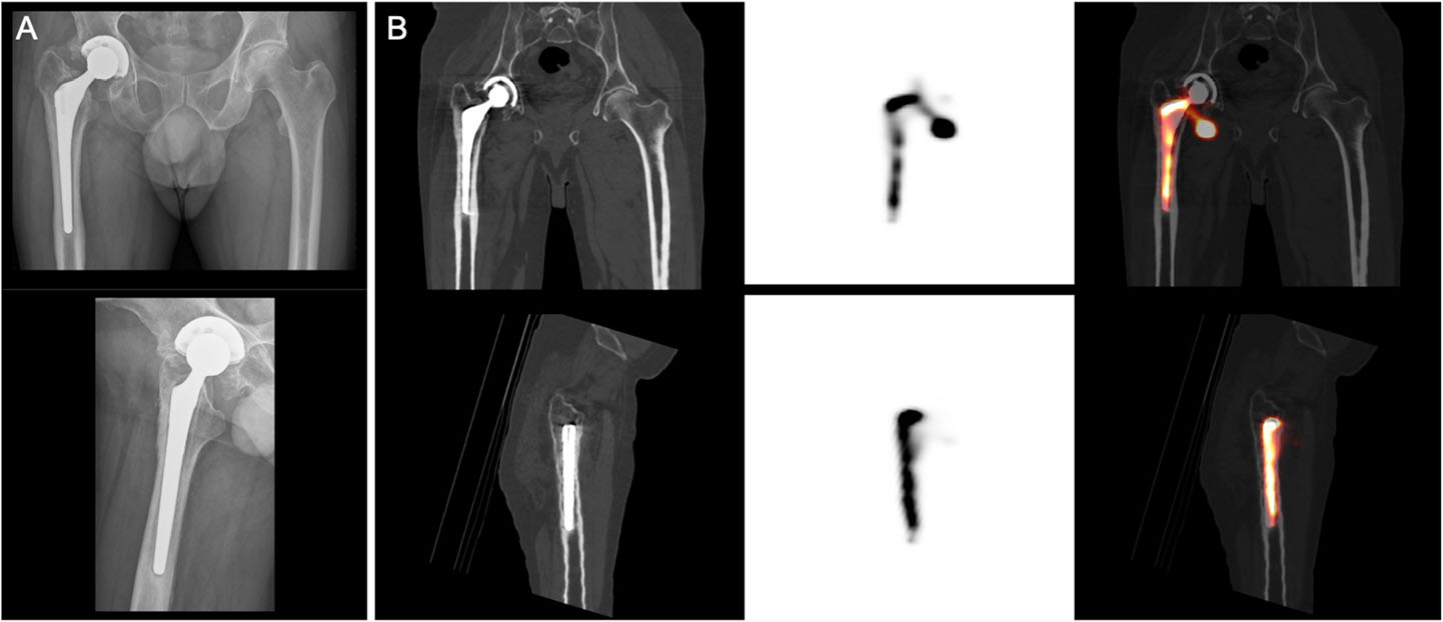
Femoral component loosening of a right hip total arthroplasty. The radiograph images demonstrate lucency along the metal-bone interface (first column). Coronal (top) and sagittal CT (second column), SPECT (third column), and fused SPECT/CT (fourth column) images showing abnormal tracer activity along the bone-prosthetic interface of the femoral component

All SPECT/CT arthrogram studies were reported by specialists licensed in nuclear medicine who were not blinded to previous imaging and clinical history. The SPECT/CT images were reviewed using Oasis workstations (Segami Corporation, Columbia, MD). SPECT/CT studies were considered negative if tracer activity was only visualized within the joint space (Fig. 2) and positive for loosening if any tracer activity was seen along the bone-prosthetic interface outside of the joint space (Figs. 3 and 4). The study was considered a failed exam if an extracapsular injection was identified on the SPECT/CT images (these patients were typically re-booked for a repeat study).

### Reference standard

Patients with persistent pain following primary arthroplasty were considered to have a loosened prosthesis if it was verified at the time of operative revision or if the patient had continued pain at 1-year follow-up with progressive radiographic features of loosening. The patients were determined not to have aseptic loosening if there was no evidence of loosening at time of operative assessment, if the pain resolved during 1-year period without revision, or if the patients were subsequently diagnosed with an alternative condition that explained their symptoms. The orthopedic surgeons were aware of the patient’s clinical history and not blinded to the SPECT/CT results during the follow-up period.

### Statistical analysis

Age of participants and time from primary arthroplasty were reported as mean ± standard deviation. Sex, anatomical location, and type of prosthesis were reported as ratios. The agreement between the SPECT/CT report and final diagnosis was calculated to determine the sensitivity, specificity, positive predictive values, negative predictive values, and diagnostic accuracy.

## Results

The baseline demographic information and type of prostheses, as well as the average time interval from primary arthroplasty procedure to SPECT/CT imaging is reported in Table 1. There were no documented adverse reactions to the SPECT/CT arthrogram procedure.

**Table 1:** Baseline demographic, timing of imaging, and type of prostheses in participants

Characteristic	Participants (***n*** = 63)
Age (years)	69 ± 10
Sex (M to F)	26:37
Anatomy (hip to knee)	27:36
Time from primary arthroplasty to imaging (months)	58 ± 51
Type of prostheses (cemented to non-cemented)	20:43

### Reference standard—surgery

In 14 of the 63 patients, revision surgery was performed following the SPECT/CT arthrogram study. Nine were knee arthroplasties and five were hip arthroplasties. For six of these patients, surgery was prompted by a positive SPECT/CT arthrogram study. A diagnosis of aseptic loosening was confirmed intraoperatively in all six patients. Surgery was also performed in eight patients with negative SPECT/CT arthrogram studies based on a high clinical suspicion despite negative imaging. In seven of these patients, the operative evaluation revealed an alternative non-loosening diagnosis. In one patient, the orthopedic surgeon diagnosed a subsiding tibial tray (false-negative SPECT/CT arthrogram study).

### Reference standard—clinical follow-up

In 49 patients, surgery was not performed. These patients were instead followed clinically with radiologic evaluation for a minimum period of 1-year post symptom onset. This group included 48 negative SPECT/CT arthrogram studies (true negative (TN)) and one positive SPECT/CT arthrogram study (false positive (FP)). Of the TN studies, 16 patients had pain which spontaneously resolved on follow-up and 32 patients had alternative diagnosis made by their orthopedic surgeon. Alternative diagnoses included trochanteric bursitis, spinal stenosis, anasarca, traumatic fracture, abscess, lumbar osteoarthritis, hematoma, referred arthritic pain, and patellofemoral pain syndrome. These patients had no further radiologic indications of loosening on their 1-year follow-up radiography. The one FP SPECT/CT arthrogram study was reported as positive for knee arthroplasty loosening; however, the pain resolved spontaneously without intervention. There was no evidence of loosening on follow-up radiography.

### Overall accuracy

Overall, this study involving 63 SPECT/CT arthrogram evaluation of clinically suspected aseptic loosening of hip and knee arthroplasty demonstrated 6 true-positive (TP), 55 TN, 1 FP, and 1 false-negative (FN) results. This results in a sensitivity of 6/7 (86%), specificity of 55/56 (98%), positive predictive value (PPV) of 6/7 (86%) and negative predictive value (NPV) of 55/56 (98%), and diagnostic accuracy of 61/63 (97%).

## Discussion

Our data confirms that SPECT/CT arthrography has a high diagnostic accuracy for detecting aseptic loosening in patients with persistent pain following primary hip and knee arthroplasties (sensitivity of 86%, specificity of 98%, PPV of 86%, NPV of 98%, and overall accuracy of 97%). Comparable results have been reported in previously published studies. In a previous retrospective study of a smaller series of 38 SPECT/CT hip and knee arthrograms from our institution (different patient cohort than included in this study), this technique was found to have a sensitivity of 100.0%, specificity of 96.0%, PPV of 92.9%, NPV of 100.0%, and overall accuracy of 97.4% for loosening (Abele et al. 2015). In another study by Murer et al*.,* examining SPECT/CT for the evaluation of loosening of total knee arthroplasties, a sensitivity of 96%, specificity of 100%, PPV of 72.7%, and NPV of 100% was reported for detecting tibial component loosening. As well, a sensitivity of 95%, specificity of 100%, PPV of 42.9%, and NPV of 100% was reported for detecting femoral component loosening (Murer et al. 2020). The study concluded that SPECT/CT helped in diagnosing aseptic loosening in patients with persistent pain after primary total knee arthroplasty.

While reported studies have consistently shown high sensitivity and specificity for this technique, our evaluation of 63 patients did demonstrate 1 FP and 1 FN case. In the FN case, while the SPECT/CT was reported as negative for loosening, the revision surgery performed 4 months after the SPECT/CT scan demonstrated a subsiding tibial tray. The report in this case did describe substantial streak artifact on the associated CT study precluding assessment of prosthetic/osseous structures (metal artifact suppression not applied). This artifact may have contributed to the false-negative result and decreased sensitivity. With metal artifact suppression techniques now available for SPECT/CT scanners, image quality may be improved, potentially reducing the impact of this artifact. In the FP case, a review of the images demonstrated mis-registration between the SPECT and CT acquisition, likely related to patient motion. With the images re-aligned, activity is seen extending into the bone-prosthetic interface of the medial condyle region; however, this does not follow the expected contour of this interface suggesting it is likely artifact.

To diagnose loosening, several imaging modalities have been described including plain radiography, bone scintigraphy, planar nuclear arthrography, SPECT/CT, 18F-FDG-PET, and/or MRI (Barnsley and Barnsley 2019). A meta-analysis by Temmerman et al. reported a pooled sensitivity and specificity for plain radiography of 82% and 81%, respectively (Temmerman et al. 2005). The most recent study on bone scintigraphy by Claassen et al*.* reported a sensitivity of 76% and a specificity of 83% (Claassen et al. 2014). Interestingly, planar nuclear arthrography on its own has a low reported sensitivity and specificity of 87% and 64% (Temmerman et al. 2005). Comparatively, we found a much higher sensitivity and specificity with SPECT/CT arthrography demonstrating the importance of the hybrid nuclear medicine imaging technique in the evaluation of aseptic loosening.

Our study has multiple limitations. First of all, as a retrospective study design, it is inherently prone to selection bias. Moreover, as the surgeons were not blinded to the SPECT/CT reports, the decision to perform the surgical revision reference standard was influenced by the outcome of the SPECT/CT diagnostic test therefore confounded by verification bias. As well, since the study involves clinical reports from multiple different readers, there is also the potential for variability in interpretation of the scans. Additionally, our study did not compare SPECT/CT arthrography results with that of SPECT/CT arthrography combined with bone scintigraphy. Further study examining both modalities could take place. Finally, while our results demonstrate a high diagnostic accuracy for our SPECT/CT arthrogram technique, it is uncertain if this technique is optimal. Specifically, a wait time of 30 min with ambulation or 60 min without ambulation may be too short. It is uncertain if a longer wait time may have impacted our FN result. There are no reported studies examining the optimal time from injection to imaging. Further study is warranted in this regard.

In summary, SPECT/CT arthrography appears to be highly accurate for the diagnosis of aseptic loosening in patients with hip or knee arthroplasties. Given this, SPECT/CT arthrography should be considered an important diagnostic adjunct, particularly for patients where there is clinical uncertainty.

## Data Availability

The datasets used during the current study are available from the corresponding author on request.

## Abbreviations

SPECT/CT: Single photon-emission computed tomography/computed tomography
18F-FDG-PET: 18F-fluorodeoxyglucose-positron emission tomography/computed tomography
MRI: Magnetic resonance imaging
CT: Computed tomography
PPV: Positive predictive value
NPV: Negative predictive value
TN: True negative
FN: False negative
TP: True positive
FP: False positive
